# Development of a computerized 2D rating scale for continuous and simultaneous evaluation of two dimensions of a sensory stimulus

**DOI:** 10.3389/fpsyg.2023.1127699

**Published:** 2023-03-03

**Authors:** Marie-Eve Hoeppli, Taylor S. Thurston, Mathieu Roy, Alan R. Light, Markus Amann, Richard H. Gracely, Petra Schweinhardt

**Affiliations:** ^1^Alan Edwards Center for Research on Pain, McGill University, Montreal, QC, Canada; ^2^Pediatric Pain Research Center, Cincinnati Children’s Hospital Medical Center, Cincinnati, OH, United States; ^3^Division of Behavioral Medicine and Clinical Psychology, Cincinnati Children’s Hospital Medical Center, Cincinnati, OH, United States; ^4^Department of Nutrition and Integrative Physiology, The University of Utah, Salt Lake City, UT, United States; ^5^Department of Psychology, McGill University, Montreal, QC, Canada; ^6^Department of Anesthesiology, The University of Utah, Salt Lake City, UT, United States; ^7^VAMC, Geriatric Research, Education, and Clinical Center, Salt Lake City, UT, United States; ^8^Department of Endodontics, School of Dentistry, Center for Neurosensory Disorders, The University of North Carolina, Chapel Hill, Chapel Hill, NC, United States; ^9^Faculty of Dentistry, McGill University, Montreal, QC, Canada; ^10^Faculty of Medicine, McGill University, Montreal, QC, Canada; ^11^Integrative Spinal Research, Department of Chiropractic Medicine, Balgrist University Hospital, Zurich, Switzerland

**Keywords:** two-dimensional scale, rating, pain, fatigue, sensory measurement, computerized scale

## Abstract

**Introduction:**

One-dimensional rating scales are widely used in research and in the clinic to assess individuals’ perceptions of sensory stimuli. Although these scales provide essential knowledge of stimulus perception, their limitation to one dimension hinders our understanding of complex stimuli.

**Methods:**

To allow improved investigation of complex stimuli, a two-dimensional scale based on the one-dimensional Gracely Box Scale was developed and tested in healthy participants on a visual and an auditory task (rating changes in brightness and size of circles and rating changes in frequency and sound pressure of sounds, which was compared to ratings on one-dimensional scales). Before performing these tasks, participants were familiarized with the intensity descriptors of the two-dimensional scale by completing two tasks. First, participants sorted the descriptors based on their judgment of the intensity of the descriptors. Second, participants evaluated the intensity of the descriptors by pressing a button for the duration they considered matching the intensity of the descriptors or squeezing a hand grip dynamometer as strong as they considered matching the intensity of the descriptors.

**Results:**

Results from these tasks confirmed the order of the descriptors as displayed on the original rating scale. Results from the visual and auditory tasks showed that participants were able to rate changes in the physical attributes of visual or auditory stimuli on the two-dimensional scale as accurately as on one-dimensional scales.

**Discussion:**

These results support the use of a two-dimensional scale to simultaneously report multiple dimensions of complex stimuli.

## 1. Introduction

Most studies investigating the perception and processing of sensory events use simple stimuli, defined as a stimulus that includes a low level of information (Naumer and Kaiser, 2010). More recently, a new approach for the investigation of sensory processing has been developed, using complex stimuli (Naumer and Kaiser, 2010). Complex stimuli, which usually require the processing of several dimensions (Faubert, 2002), have the advantage that they are more similar to everyday life sensory events and convey a higher amount of information than simple stimuli (De Gelder and Bertelson, 2003; Vatakis and Spence, 2006; Naumer and Kaiser, 2010). Because of their relevance to understand ecologically valid perceptual processes, complex stimuli are increasingly being used in research (Allen and Oxenham, 2014).

Studies using complex stimuli mostly manipulate one stimulus dimension to investigate the effect of changing one dimension on the perception of another dimension (Garner, 1976; Melara and Marks, 1990b; Neuhoff et al., 2002; Naumer and Kaiser, 2010; Walker and Walker, 2012; Walker et al., 2015). For example, Walker et al. (2015) showed that the brightness of visual stimuli interacts with the perception of the size of the stimuli. In fact, for some complex stimuli, it might even not be possible to experimentally manipulate one dimension without potentially inducing changes in the perception of another dimension. Assessing two dimensions of a complex stimulus would allow to capture the perception of this stimulus more completely, as well as potential interactions of the dimensions. The assessment of the perception of two stimulus dimensions in parallel has been previously reported in the literature. Such assessment typically includes multiple scales presented in alternating sequence (Kerrick et al., 1969; Price et al., 1983), allowing for discrete ratings of each dimension. However, in some instances it would be advantageous to acquire continuous ratings, especially when perceptions or sensations fluctuate over time, such as pain or fatigue sensations (Suzan et al., 2015). Measuring these sensations are the underlying reason for the development of this scale. Thus, acquiring continuous two-dimensional ratings would be important for the study of some forms of perceptual processing but, to the best of our knowledge, two dimensions have not yet been continuously rated on a single scale.

Therefore, the aim of this study is to assess the ability of healthy individuals to rate continuously two dimensions of the same stimulus using a two-dimensional rating scale (2D), based on the one-dimensional Gracely Box Scale (Gracely and Dubner, 1987). To this end, stimuli of which the physical properties can be well controlled, i.e., auditory and visual stimuli, were used to assess participants’ ability to rate on a 2D scale.

## 2. Materials and methods

### 2.1. Participants

To ensure that the 2D scale was an appropriate rating tool for adults of various ages, 17 younger healthy volunteers (10 males and 7 females; whole sample age mean ± SD: 27.1 ± 3.2; whole sample age range: 23–33 years) and 15 older healthy volunteers (9 males and 6 females; whole sample age mean ± SD: 68.8 ± 4.8; whole sample age range: 62–76 years) were enrolled in this study, resulting in a total sample of 32 participants. Exclusion criteria were age below 18 years, history of neurological or psychiatric disorder, medication or recreational drugs, any serious pathology, any diagnosed hearing deficit, any uncorrected visual deficit. Written informed consent was obtained from each participant prior to the beginning of the study. All experimental procedures were approved by the Institutional Review Boards of The University of Utah and of the Salt Lake City Veteran’s Affairs Medical Center and conducted according to the Declaration of Helsinki for human experimentation.

### 2.2. Two-dimensional scale

The 2D scale was adapted from the one-dimensional Gracely Box Scale (Gracely and Dubner, 1987), which uses a logarithmic distribution of the descriptors along the axis. The logarithmic distribution allows accurate ratings of low sensations, which are undersampled using a linear scale. Given that some somatosensory stimuli might induce subtle sensations or changes in sensations, a rating tool that allows accurate rating of weak sensations is essential. Participants rate their sensations using 13 descriptors (‘no sensation,’ ‘faint,’ ‘very weak,’ ‘weak,’ ‘very mild,’ ‘mild,’ ‘moderate,’ ‘barely strong,’ ‘slightly intense,’ ‘strong,’ ‘intense,’ ‘very intense,’ ‘extremely intense’). The Gracely Box Scale has been validated for rating the intensity and unpleasantness of painful sensations (Gracely and Dubner, 1987). The 2D scale is developed to rate these sensations along with fatigue sensations. Because it is inherently difficult to control the stimulus intensity of continuous pain and fatigue sensations, auditory and visual stimuli that allow better control of the physical properties of the stimuli, were used to confirm participants’ ability to rate on the 2D scale. Usage of this scale in the context of pain and fatigue sensations induced by intra-muscular physiological infusions of a mix of ATP, lactate, and proton is described in a separate article (Hoeppli et al., in preparation).

### 2.3. General design

First, participants performed two tasks to assess the understanding of the descriptors displayed on the scales. Second, participants performed a visual task on the 2D scale. Third, participants completed an auditory task to compare their ability to rate on the 2D scale with their ability to rate on 1D scales. All participants completed these tasks in the same order.

### 2.4. Descriptors and scale development tasks

Two tasks were used to assess how participants evaluated the perceived intensity of the scale descriptors. These tasks were part of the original experiment to validate the Gracely Box Scale (Gracely and Dubner, 1987) and were included here to ensure that (i) the ranking of the descriptors provided by the participants of the present study was comparable to the one in the original study and (ii) rank order and logarithmic magnitude estimation of descriptors are similar for pain and fatigue sensations, which has not previously been tested for the Gracely Box Scale.

#### 2.4.1. Descriptor task 1: Descriptor ranking

Participants were asked to rank the pain and fatigue descriptors that were used on each axis from the least intense to the most intense. The descriptors were written on cards with one set of cards for fatigue and one set of cards for pain. Each card of one set displayed one of the intensity descriptors mentioned above and the word ‘fatigue’ or ‘pain.’ Participants were instructed that there was no predefined category and that they should rank the descriptors as they deemed appropriate. The order of the set of cards, i.e., fatigue or pain, was counterbalanced between participants.

#### 2.4.2. Descriptor task 2: Descriptor magnitude estimation

To further assess the perceived intensity represented by each descriptor, all participants were asked to estimate the magnitude/intensity of three different types of stimuli: the intensity of fatigue descriptors (number of stimuli: 12), the intensity of pain descriptors (number of stimuli: 12) and the length of lines (number of stimuli: 7). The stimuli were displayed on a computer screen and controlled by the software Presentation (version 17.2, Neurobehavioral Systems, Berkeley, CA, USA). Two response modalities were used for magnitude estimation of each stimulus to ensure that results were not modality-specific. For one response modality, participants squeezed an electronic handgrip dynamometer as strongly as they perceived the magnitude/intensity of the stimuli (i.e., the squeeze would be stronger for a line perceived as longer compared to a line perceived as shorter). For the second response-modality, participants evaluated the stimuli by pressing a button for the amount of time they judged to correspond to the magnitude/intensity of the stimulus (i.e., the longer they pressed the button, the higher they found the magnitude/intensity of the stimulus). The order of the two response-modalities was counterbalanced across participants.

Participants first evaluated the length of lines. This allowed them to train both response modalities with a simple stimulus. In addition, the individual’s evaluation of the length of the lines was used to define individual calibration curves used to estimate the magnitude of the intensity descriptors at a group level. Then, participants were asked to evaluate the intensity of the pain and fatigue descriptors. Each participant evaluated each descriptor three times with both methods (handgrip and button press). The order of the sensations, i.e., pain and fatigue, was counterbalanced between participants. Peak strength during the handgrip response modality was recorded using a hand dynamometer connected to a Biopac system (Goleta, CA, USA). Button press duration was recorded by the Presentation software.

#### 2.4.3. Scale development visual task

Participants were presented with circles that changed in brightness and size on a computer screen using the Presentation software. There were four conditions in this task: the circles could vary in one, both or neither dimension(s). Changes in either dimension were independent from changes in the other dimension. The size of the circles ranged from 20 pixels to 300 pixels. The brightness of the circles was defined based on their color from white (brightest) to black (darkest). It was defined in RGB system and ranged from (245, 245, 245) to (0, 0, 0).

**FIGURE 1:**
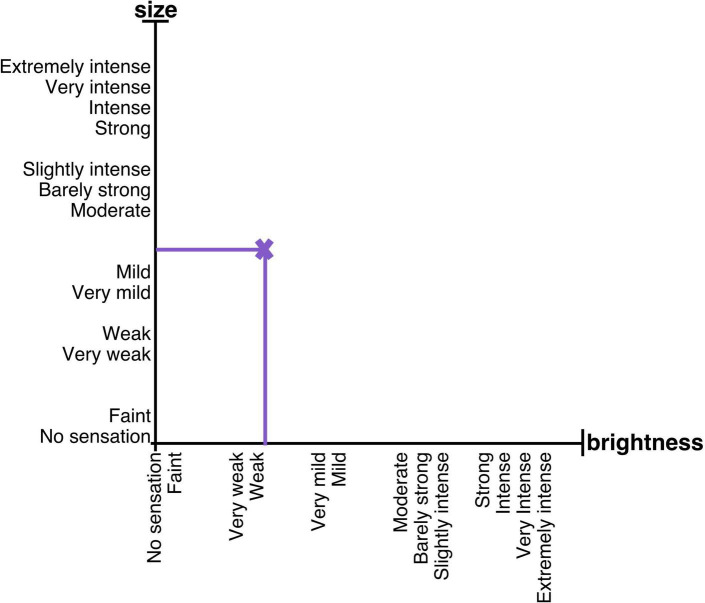
2D scale displayed during the visual task. During the visual task, participants were instructed to rate any changes in size or brightness of circles on the 2D scale. The assignment of x- and y-axes to size or brightness was counterbalanced across participants. Given that the results of the descriptors and scale development tasks described here matched the order of the descriptors on the Gracely Box Scale, the order of the descriptors replicated their order on the Gracely Box Scale.

Participants were asked to rate 20 potential changes in dimensions, i.e., 5 changes per condition. The timing of each change was pseudorandomized. The overall duration of the task was 3 min and 15 s. The duration of the individual circles ranged from 6,742 milliseconds to 13,103 milliseconds. Participants were instructed to continuously rate changes in the physical attributes of the circle, i.e., size and brightness, by moving a cross on the 2D scale (Figure 1) with a trackball mouse (Trackman Marble, Logitech, Newark CA, USA). The 2D scale was displayed alongside the circles on a computer screen using the Presentation software. Ratings were automatically recorded by the Presentation software at each screen refresh, approximately every 20 milliseconds.

One axis of the scale allowed ratings of size, while the other allowed ratings of brightness. The descriptors were displayed on each axis of the scale. Because the descriptors do not fit a description of size, participants were instructed to consider the axis as representing a magnitude; the bigger the circle was perceived, the more the cross should be moved toward the ‘extremely intense’ descriptor. The assignment of x- and y-axes to ratings of size or brightness was counterbalanced across participants.

#### 2.4.4. Scale development auditory task

**FIGURE 2:**
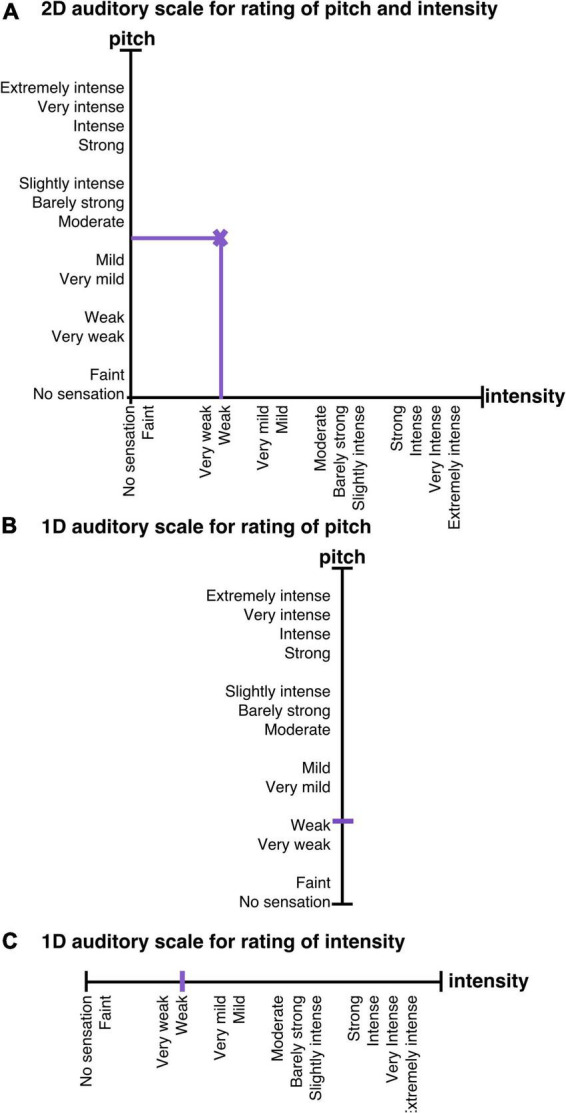
Scales displayed during the auditory task. In the auditory task, participants completed three parts. During the first part, they were instructed to rate changes in volume and pitch (frequency) of sounds on a 2D scale **(A)**. During the second part, participants rated changes in pitch (frequency) of the sounds on a 1D scale **(B)**. During the third part, participants reported perceived changes in the volume of the sounds on a 1D scale **(C)**. The order of the parts and the assignment of x- and y-axes to pitch or volume were counterbalanced across participants.

For each part, participants were instructed to rate on one of the three following scales: a 2D scale (Figure 2A) that displayed one axis to rate changes in volume (perceived sound pressure level) and one axis to rate changes in pitch (perceived frequency), a 1D scale to rate changes in pitch (Figure 2B), and a 1D scale to rate changes in volume (Figure 2C). All axes displayed all the descriptors with which the participants were previously familiarized. The 1D scales were exact copies of the corresponding axis of the 2D scale. Scales were displayed on a computer screen using the Presentation software. Ratings were automatically recorded by the Presentation software at each screen refresh, approximately every 20 milliseconds.

*Via* Technics Stereo over-ear headphones (Panasonic Corporation, Newark, NJ, USA), participants were presented with sounds that changed in sound pressure level (i.e., volume) and frequency. This task included four conditions and three parts with different rating scales. The four conditions were the following: the sounds could vary in one, both or neither physical attribute(s). Changes in either dimension were independent from changes in the other dimension. Each part included all the conditions. Participants underwent 24 changes per part, i.e., 6 changes per condition. The duration of the sounds was pseudorandomized (mean: 8 s; sd: 1.45 s). The duration of one part was between 3 min 9 s and 3 min 16 s. The duration of the individual sounds ranged from 5,600 milliseconds to 10,500 milliseconds.

Three sets of sounds were used and randomized between the parts to avoid any learning effects. The frequency of the sounds ranged from 100 Hertz to 1,500 Hertz. The volume was individually adjusted to ensure that all participants could hear the sounds clearly. The program was then set to adjust the volume by applying an attenuation ranging from 15 decibels to 65 decibels.

Similarly to size in the visual task, participants were instructed to evaluate the sound’s frequency as a magnitude; the higher the perceived frequency (i.e., pitch), the closer to the ‘extremely intense’ descriptor participants were to move the cross. Participants were asked to rate continuously on the 1D and 2D scales using the same trackball mouse as previously. The assignment of x- and y-axes to volume or pitch as well as the order of the scales was counterbalanced across participants.

### 2.5. Statistical analysis

#### 2.5.1. Analysis of the descriptor ranking task

To analyze the ‘descriptor ranking’ task, the percentage of participants classifying each descriptor at the same rank as in the original study (Gracely and Dubner, 1987) was calculated.

#### 2.5.2. Analysis of the descriptor magnitude estimation task

Two analyses of the ‘descriptor magnitude estimation’ task were performed:

1. Peak handgrip strength and duration of the button press were averaged across the three trials of each stimulus, i.e., each line, each fatigue descriptor and each pain descriptor. Multilevel regressions (using the software Hierarchical Linear and Nonlinear Modeling HLM7, Scientific Software International Inc., Skokie, IL, USA) were used to investigate whether the intensity of the stimuli predicted the handgrip strength or button press duration provided by the participants. Multilevel regressions are used when data are organized at more than one level. The first level characterizes within-subject and individual predictors, while higher levels define group predictors (Woltman et al., 2012; Tabachnick and Fidell, 2013). Age (two groups: younger and older) was defined as a group predictor in the regression models to test whether age influenced the perception of the magnitude of the descriptors. To investigate any effect of the intensity (i.e., line length or the rank of the descriptors) and type of the stimuli, and age on handgrip strength or button press duration, two three-level regressions were modeled, one for each response modality [handgrip strength or button press duration as dependent variables; first-level predictor: intensity of the stimuli; second-level predictor: stimuli type (lines, fatigue descriptors, and pain descriptors); third-level predictor: age group]. In addition, to investigate the effect of the intensity of the stimuli and age on button press duration and handgrip strength within each type of stimulus and response modality, one model was defined for each response modality (hand grip strength or button press duration) and for each stimulus type (lines, fatigue descriptors, and pain descriptors), resulting in six models of two-level regressions (first-level predictor: intensity of the stimuli of the three stimulus types; second-level predictor: age group). In all regression models, the intensity of the stimuli was defined as the rank of the descriptors from the original study (Gracely and Dubner, 1987) (‘no sensation,’ ‘faint,’ ‘very weak,’ ‘weak,’ ‘very mild,’ ‘mild,’ ‘moderate,’ ‘barely strong,’ ‘slightly intense,’ ‘strong,’ ‘intense,’ ‘very intense,’ ‘extremely intense’).
2. The second analysis replicated the original analysis of this task, as described in details in Gracely et al. (1978). In brief, geometric means of handgrip strength, respectively button press duration, were calculated and standardized within subject, condition and stimulus. Power exponents were calculated for each modality in the line condition and used to calculate the relative magnitude of the descriptors.

#### 2.5.3. Analysis of the scale development visual and auditory tasks

Ratings recorded during the visual and auditory tasks were downsampled offline to a rate of 250 milliseconds.

Two two-level linear models were defined for the analyses of the visual task and inputted in HLM7. The first model included the ratings of perceived size as the dependent variable and three first-level predictors, i.e., the physical values of size and brightness of the circles and the physical value of the preceding circle’s size. The size of the preceding circle was entered as a predictor because stimulus perception has been shown to be influenced by physical attributes of the previous stimulus and the difference to the physical attribute of the current one (Smoorenburg, 1970; Snyder et al., 2009). The second level defined the ‘age group’ predictor to test whether age affected the ability to rate on a 2D scale. The second model was identical except that the ratings of perceived brightness served as dependent variable, and the first-level predictor of the physical attribute of the preceding circle was the previous circle’s brightness.

For the auditory task, two three-level regressions were first performed to assess whether the different sets of sounds, which were used in each part, influenced the results [ratings of pitch (or volume) as dependent variable; first-level predictor: physical values of frequency and sound pressure level, physical values of the frequency (or sound pressure level) of the preceding sounds; second-level predictor: set of sounds; third-level predictor: age group]. The sets of sounds did not have a significant influence on the ratings of pitch and volume. Therefore, this predictor was omitted in the final two three-level regressions that were performed to assess the effects of the different scales and of the age group on the ratings of pitch and volume [ratings of pitch (or volume) as dependent variable; first-level predictor: physical values of frequency and sound pressure level, physical values of the frequency (or sound pressure level) of the preceding sounds; second-level predictor: scale type (1D or and 2D scale); third-level predictor: age group].

For all analyses, significance levels were set at 5%. *P*-values above 0.05 but below 0.1 were considered as trend (Bangalore and Messerli, 2006).

## 3. Results

### 3.1. Descriptor and scale development tasks

#### 3.1.1. Descriptor task 1: Descriptor ranking

**FIGURE 3:**
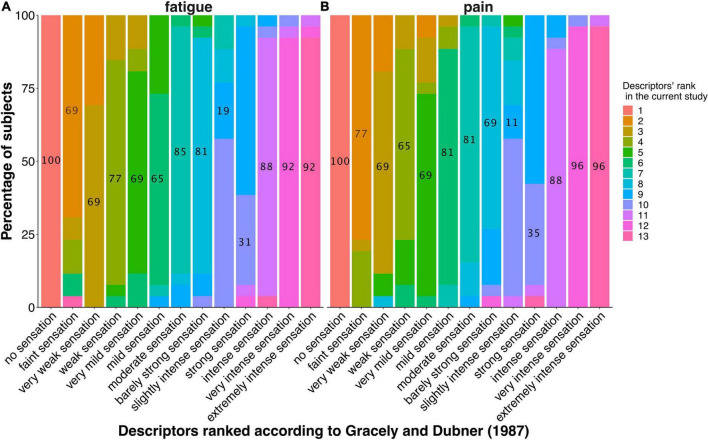
Ranking of the intensity descriptors during the familiarization task of ranking of the fatigue descriptors **(A)** and pain descriptors **(B)**. The graphs display the percentage of participants in each rank. The descriptors are organized on the x-axis in the order described in Gracely and Dubner (1987). Labels on each bar represent the percentage of participants ranking the respective descriptor at the same rank as in Gracely and Dubner (1987), showing that most descriptors were ranked exactly in the same order as in the original study.

Pain (Figure 3A) and fatigue (Figure 3B) descriptors were largely ranked in the same order as in the original validation study of the Gracely Box Scale (Gracely and Dubner, 1987) by the majority of participants. Only two descriptors (‘slightly intense’ and ‘strong’ for pain as well as fatigue) were rank-exchanged in slightly more than 50% of the participants.

#### 3.1.2. Descriptor task 2: Descriptor magnitude estimation

The multilevel regression analysis of the descriptor magnitude estimation task showed that participants evaluated increasing intensities of the pain and fatigue descriptors with increasing strength in the handgrip task and increasing button press duration. Specifically, the two three-level regressions showed a significant effect of stimulus intensity (i.e., line length, intensity of pain and fatigue descriptors) on the handgrip strength and button press duration (handgrip strength: t-ratio = 11.63, *p* < 0.001, percentage of variance explained = 66%; button press duration: t-ratio = 5.938, *p* < 0.001, percentage of variance explained = 38%). Both regressions revealed that the type of stimulus (line, fatigue, and pain) influenced the effect of the stimulus intensity on the handgrip strength and button press duration (handgrip strength: t-ratio = −4.48, *p* < 0.001; button press duration: t-ratio = 3.583, *p* < 0.001). To test whether the intensity of the stimuli has an effect on handgrip strength and button press duration for each type of stimulus, six two-level regressions were performed, one for each type of stimuli and response. These analyses showed a significant effect of intensity on the handgrip strength or button press duration for each type of stimulus (Table 1). There was no significant effect of age on the effect of intensity on the applied handgrip strength or button press duration.

**TABLE 1:** Effect of the intensity of stimuli on applied handgrip strength and button press duration for each stimulus type.

	Handgrip strength		Button press duration	
Stimulus type	T-ratio	*P*-value	T-ratio	*P*-value
Lines	11.734	<0.001	5.506	<0.001
Fatigue descriptors	10.4	<0.001	5.764	<0.001
Pain descriptors	9.899	<0.001	6.445	<0.001

The analysis performed following the method described in the original articles (Gracely et al., 1978; Gracely and Dubner, 1987) supported these findings, showing overall an increased relative magnitude with increasing intensity of the descriptors of fatigue or pain (Table 2). There was no significant difference between the relative magnitude of fatigue descriptors and pain descriptors [*t*_(11)_ = −1.38, *p* = 0.19]. Unexpectedly, the relative magnitude of the fatigue descriptor “barely strong” was slightly greater than the one of the fatigue descriptors of lesser intensity “moderate” (1.1095, resp. 1.1099). Similarly, the relative magnitude of the pain descriptor “very weak” was greater than the one of the pain descriptor of lesser intensity “faint” (0.37, resp. 0.43).

**TABLE 2:** Relative magnitudes of intensity descriptors for fatigue and pain.

Intensity	Fatigue relative magnitude	Pain relative magnitude
Faint	0.4419	0.426
Very weak	0.4435	0.3699
Weak	0.5402	0.5032
Very mild	0.5514	0.53
Mild	0.7484	0.752
Moderate	1.1099	1.1484
Barely strong	1.1094	1.2524
Slightly intense	1.2076	1.2856
Strong	1.6716	1.6476
Intense	1.7502	1.8163
Very intense	2.1735	2.196
Extremely intense	2.4191	2.6018

The table displays the relative magnitudes for each intensity descriptor of fatigue and pain sensations. These relative magnitudes were calculated following the methods described in Gracely and Dubner (1987).

The results of the two descriptor tasks show that: (1) the ranking of the descriptors in the present study largely overlaps with the one from the original study; (2) the intensity of the descriptors from the original study is highly predictive of our participants’ responses, i.e., handgrip strength or button press duration. These results support using the same order of the descriptors as originally described.

#### 3.1.3. Scale development visual task

##### 3.1.3.1. Rating of the circle’s size

**FIGURE 4:**
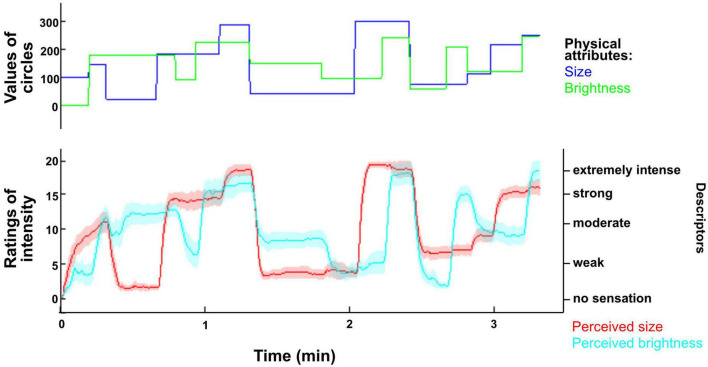
Time course of ratings of size and brightness in the visual task. The upper panel of the plot displays the time course of the changes in the physical attributes (blue: size; green: brightness) of the circle. The lower panel shows the time course of the average ratings of perceived size and brightness on the 2D scale with a 95% confidence interval (red: size; cyan: brightness). The time course of the ratings suggests that participants adapted their ratings appropriately following changes in the physical attributes of the circle.

Figure 4 depicts the time courses of the size ratings of the circle. This graph indicates that participants were able to follow the physical changes of the circle’s size with their ratings. The results of the multilevel regression for the same dimension (Table 3) show that the three predictors, i.e., physical value of size, physical value of brightness, and physical value of the preceding circle’s size, each had a significant effect on the ratings of perceived size. The positive coefficient of the physical value of the circle’s size indicates that the bigger the size of the circle, the higher the rating of size is. Similarly, the coefficient of the preceding circle’s size indicates that ratings of size increase when the preceding circle was bigger than the current circle. Finally, the negative coefficient related to the circle’s brightness indicates that brighter circles were judged as being smaller. The physical value of the circle’s size had the strongest effect on the size ratings, as indicated by the highest t-ratio of 74.085, and accounted for 79% of the variance in size ratings. This effect is approximately five times the effect of the preceding circle’s size and ten times the effect of brightness. The age group of the participants did not significantly impact the effect of the physical value of the circle’s size or the effect of the preceding circle’s size. However, age had a significant, albeit small, impact on the effect of the physical value of brightness (Table 3): compared to the younger participants, older participants rated brighter circles as bigger.

**TABLE 3:** Effect of circle attributes and age group on the ratings of the size of the circle.

	Coefficient	Standard error	T-ratio	*P*-value
Intercept	9.82	0.158	61.999	<0.001
Effect of age	-0.43	0.277	-1.555	n.s.
Physical value of size	0.056	0.0007	74.085	<0.001
Effect of age	-0.001	0.001	-0.804	n.s.
Physical value of brightness	-0.004	0.0006	-7.529	<0.001
Effect of age	0.0026	0.001	2.184	<0.05
Previous circle’s physical value of size	0.009	0.0006	15.289	<0.001
Effect of age	0.0005	0.001	0.36	n.s.

The multilevel regression shows a significant effect of the physical size or brightness of a circle on the rating of its size, as well as a significant effect of the physical size of the previous circle. The strongest predictor of the rating was the physical size of a circle. In addition, age group had a significant effect on the physical brightness of a circle as predictor.

##### 3.1.3.2. Rating of the circle’s brightness

Similar to size, participants were able to follow changes in the physical value of brightness of the circle using the 2D scale (Figure 4). This is supported by the results of the multilevel regression for the ratings of brightness (Table 4). The strongest predictor of the ratings of perceived brightness was the physical value of the circle’s brightness; 49% of the variance in ratings of perceived brightness were accounted for by the physical value of the circle’s brightness. The physical value of brightness of the preceding circle had also a significant effect on the ratings; ratings were higher when the preceding circle was brighter. This effect was approximately three times smaller than the effect of the physical value of brightness of the current circle. The physical value of the other dimension, i.e., size, had the smallest impact on the ratings (approximately 10 times smaller than the effect of the physical value of brightness); bigger circles increased the ratings of brightness. Age group had no impact on the effect of the current or preceding stimulus’ brightness but impacted the effect of size on the ratings of brightness with older participants rating bigger circles as brighter (Table 4).

**TABLE 4:** Effect of circle attributes and age group on the ratings of brightness.

	Coefficient	Standard error	*T*-ratio	*P*-value
Intercept	11.789	0.1643	71.731	<0.001
Effect of age	-0.564	0.287	-1.971	n.s.
Physical value of brightness	0.07	0.0018	39.478	<0.001
Effect of age	-0.009	0.0056	-1.638	n.s.
Physical value of size	0.0018	0.0005	3.358	<0.01
Effect of age	0.0035	0.0012	2.965	<0.01
Previous circle’s physical value of size	0.0146	0.0011	13.348	<0.001
Effect of age	0.0009	0.0021	0.442	n.s.

The multilevel regression results show a significant effect of the physical brightness of a circle and of the previous circle on the ratings, as well as a significant effect of the physical size of a circle. The strongest predictor of the rating was the physical brightness of a circle. In addition, age group had a significant effect on the physical size of a circle as predictor.

#### 3.1.4. Scale development auditory task

**FIGURE 5:**
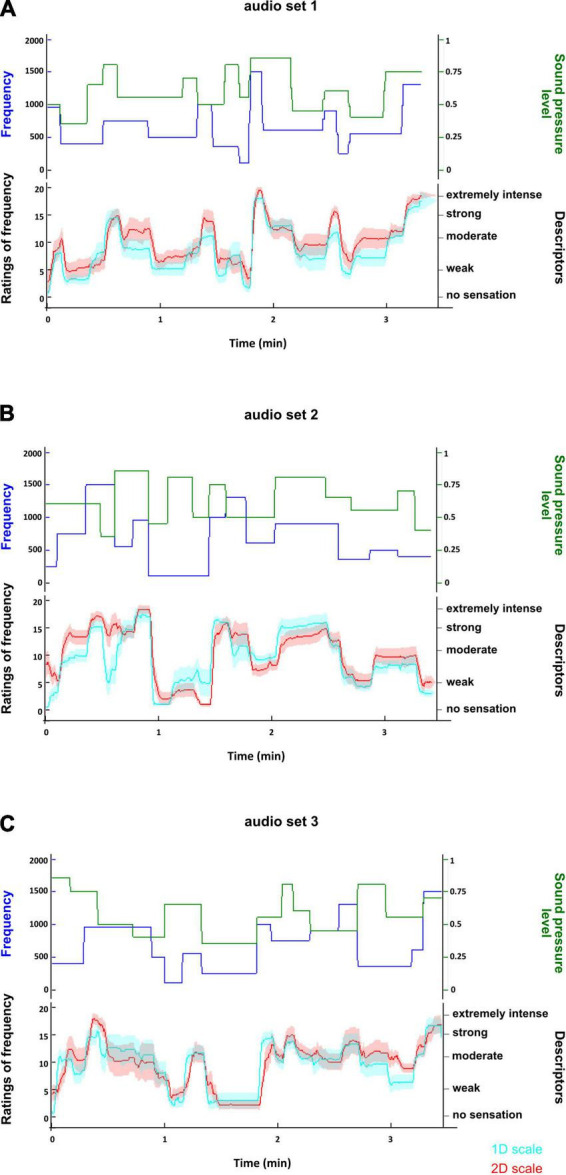
Time course of ratings of pitch of the sounds for each set of sounds used in the auditory task. **(A)** Shows the time course of the ratings in the first set of sounds; **(B)** shows the time course of the ratings in the second set of sounds; **(C)** shows the time course of the ratings in the third set of sounds. In each plot, the upper panel displays the time course of the changes in the physical attributes (frequency and sound pressure level) of the sounds. The lower panel shows the time course of the average ratings of pitch with a 95% confidence interval on the 1D scale (cyan) and on the 2D scale (red). The time courses of the ratings suggest that participants rated changes in the frequency of the sounds appropriately. Furthermore, the similarity between the time course of the 1D and 2D ratings suggests that the type of scale did not impact on the participants’ ability to rate.

**FIGURE 6:**
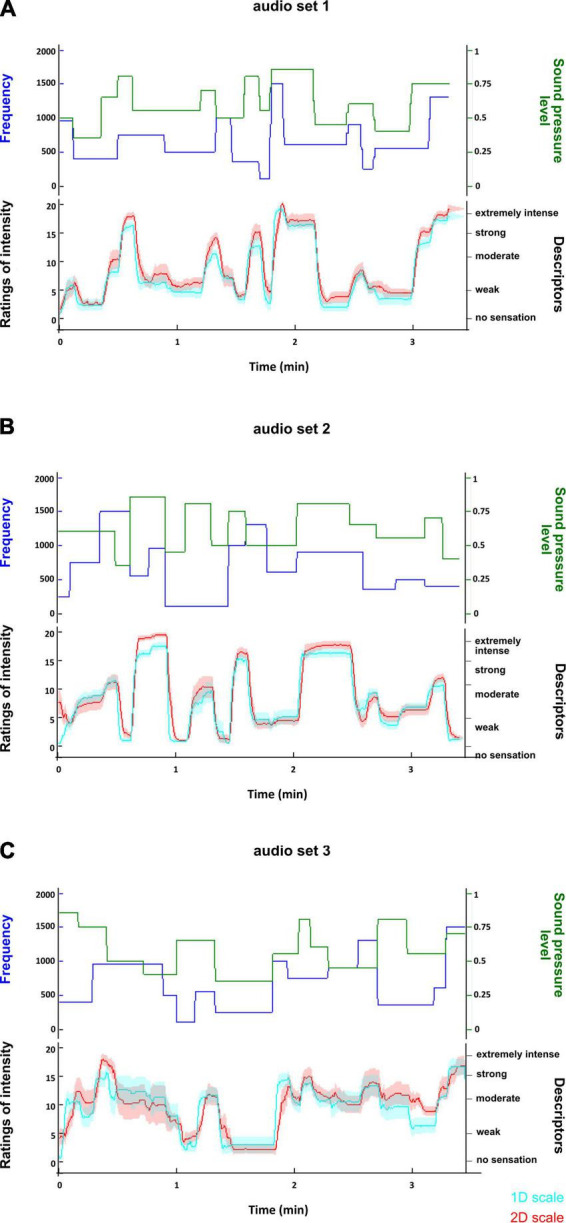
Time course of ratings of volume of the sounds for each set of sounds used in the auditory task. **(A)** Shows the time course of the ratings in the first set of sounds; **(B)** shows the time course of the ratings in the second set of sounds; **(C)** shows the time course of the ratings in the third set of sounds. In each plot, the upper panel displays the time course of the changes in the physical attributes (frequency and sound pressure level) of the sounds. The lower panel shows the time course of the average ratings of volume with a 95% confidence interval on the 1D scale (cyan) and on the 2D scale (red). The time courses of the ratings suggest that participants rated changes in the volume of the sounds appropriately. Furthermore, the similarity between the time course of the 1D and 2D ratings suggests that the type of scale did not impact on the participants’ ability to rate.

Similarly to the visual task, the time courses of participants’ ratings (Figures 5, 6) indicate that they were able to rate changes in sound pressure level and pitch of sounds on 1D and 2D scales in an accurate manner. The sets of sounds did not have any impact on the ratings of pitch and intensity (Tables 5, 6), thus it was proceeded to test whether the type of scale, i.e., 1D or 2D scales, had an impact on the ratings. The type of scale was not found to have a significant effect on the ratings of pitch or intensity (Tables 7, 8), indicating that participants were similarly able to rate on the 2D scale as on the 1D scales.

**TABLE 5:** Effect of the sets of sounds on the effect of sound attributes on the ratings of pitch.

	Coefficient	Standard error	*T*-ratio	*P*-value
Intercept	7.702	0.935	8.239	<0.001
Effect of sounds’ set	-0.997	0.905	-1.101	n.s.
Physical value of frequency	0.0067	0.0009	6.97	<0.001
Effect of sounds’ set	-0.0005	0.0009	-0.56	n.s.
Physical value of sound pressure level	4.897	1.524	3.213	<0.01
Effect of sounds’ set	-0.591	1.608	-0.367	n.s.
Previous circle’s physical value of frequency	0.00004	0.0008	0.048	n.s.
Effect of sounds’ set	0.0004	0.0007	0.557	n.s.

Results of the multilevel regression showed no significant effect of the set of sounds on the effect of physical attributes of the sounds.

**TABLE 6:** Effect of the sets of sounds on the effect of sound attributes on the ratings of volume.

	Coefficient	Standard error	T-ratio	*P*-value
Intercept	6.586	0.836	7.878	<0.001
Effect of sounds’ set	-1.086	0.81	-1.341	n.s.
Physical value of sound pressure level	17.981	1.775	10.131	<0.001
Effect of sounds’ set	-0.0622	2.551	-0.025	n.s.
Physical value of frequency	0.0025	0.003	8.092	<0.001
Effect of sounds’ set	0.00005	0.0003	0.193	n.s.
Previous circle’s physical value of sound pressure level	-1.717	1.302	-1.32	n.s.
Effect of sounds’ set	1.024	0.8	1.278	n.s.

Results of this multilevel regression showed no significant effect of the set of sounds on the effect of physical attributes of the sounds.

**TABLE 7:** Effect of scale type and age group on the effect of the physical attributes of the sounds on the ratings of pitch.

	Coefficient	Standard error	T-ratio	*P*-value
Intercept	10.328	0.431	23.983	<0.001
Effect of age	-0.532	0.653	-0.815	n.s.
Effect of scale type	-1.116	0.661	-1.689	n.s.
Effect of age	0.438	0.82	0.534	n.s.
Physical value of frequency	0.0096	0.001	9.448	<0.001
Effect of age	-0.0037	0.0015	-2.424	<0.05
Effect of scale type	-0.0002	0.0008	-0.243	n.s.
Effect of age	-0.00006	0.001	-0.045	n.s.
Physical value of sound pressure level	4.637	1.46	3.175	<0.01
Effect of age	4.667	3.719	1.255	n.s.
Effect of scale type	4.275	2.23	1.917	n.s.
Effect of age	1.127	3.69	0.305	n.s.
Previous sound’s physical value of frequency	0.0016	0.0008	1.878	n.s.
Effect of age	-0.0003	0.0013	-0.271	n.s.
Effect of scale type	-0.002	0.0014	-1.534	n.s.
Effect of age	0.0018	0.0017	1.056	n.s.

Results of the multilevel regression showed significant effects of all the predictors, including the frequency of a sound and of the previous sound and the sound pressure level of a sound, on the ratings of pitch. The strongest predictor of the rating was the frequency of a sound. Age group had only a small significant effect on one predictor, i.e., the frequency of a sound. The type of scale used, i.e., 1D or 2D scales, did not have any effect on the predictors.

**TABLE 8:** Effect of scale type and age on the effect of the physical attributes of the sounds on the ratings of volume.

	Coefficient	Standard error	T-ratio	*P*-value
Intercept	8.385	0.353	23.777	<0.001
Effect of age	0.255	0.499	0.512	n.s.
Effect of scale type	-0.908	0.545	-1.667	n.s.
Effect of age	-0.507	0.808	-0.627	n.s.
Physical value of sound pressure level	25.951	1.264	20.536	<0.001
Effect of age	-2.756	2.36	-1.168	n.s.
Effect of scale type	1.789	1.069	1.674	n.s.
Effect of age	-0.015	2.4	-0.006	n.s.
Physical value of frequency	0.0037	0.0005	7.753	<0.001
Effect of age	-0.0004	0.0007	-0.54	n.s.
Effect of scale type	0.0004	0.0006	0.731	n.s.
Effect of age	-0.001	0.0008	-1.212	n.s.
Previous sound’s physical value of sound pressure level	0.872	1.41	0.618	n.s.
Effect of age	0.516	2.429	0.212	n.s.
Effect of scale type	0.626	1.14	0.549	n.s.
Effect of age	-1.795	2.179	-0.824	n.s.

Results of the multilevel regression showed significant effects of the sound pressure level and frequency of a sound on the ratings of volume. The strongest predictor of the rating was the sound pressure level of a sound. Age group and the scale used, i.e., 1D or 2D scales, had no significant effect on the effect of the predictors (sound pressure level, frequency, and sound pressure level of the previous sound).

As for the visual task, the physical values of the respective dimension (frequency or sound pressure level) were the strongest predictors of the ratings (frequency explained 33% of the variance in pitch ratings, sound pressure level explained 49% of the variance in volume ratings). The ratings of the other dimension also had a significant effect on the ratings but in contrast to the visual task, the physical attributes of the preceding stimulus had no effect on the ratings (Tables 7, 8). Age impacted the effect of frequency on the ratings of pitch, in the sense that older participants rated high frequency sounds lower compared to younger participants.

## 4. Discussion

The results of this study indicate that healthy volunteers were able to use the 2D rating scale to rate two dimensions simultaneously and continuously. In the visual task, changes in the physical values of brightness or size largely explained the variance of the respective ratings. Similarly, changes in the physical values of frequency or sound pressure level of a sound were the main predictors of the respective ratings, suggesting that these changes were correctly rated on the 2D scale. Importantly, the auditory task showed that the rating accuracy did not differ between the 2D scale and the 1D scales. Further, there was no difference between younger and older participants in their ability to rate on the 2D or 1D scale. In addition, the original validation tasks of the Gracely Box Scale (Gracely and Dubner, 1987) were used to test whether the participants evaluated the scale descriptors in a manner comparable to the original, and never repeated, study. The results showed that the present participants ranked the pain descriptors very similarly to the participants in the original study and that the magnitude estimations correctly reflected the intensity of the descriptors. Taken together, the results of the present study support the use of the descriptors in the same order as in the original study for simultaneous ratings of pain and fatigue on a 2D scale.

Ecologically valid stimuli characteristically vary in multiple dimensions continuously and simultaneously. While individuals can easily process such multivariate stimuli, it had not been tested whether individuals can concurrently provide accurate explicit assessments of more than one dimension. Thus far, research investigating complex stimuli has typically used one or multiple 1D scales administered sequentially to assess participants’ perception (Kerrick et al., 1969; Price et al., 1983). While this approach allows evaluating more than one dimension, it only provides ‘snapshots’ of perception at discrete points in time. 1D continuous rating scales have been used to obtain uninterrupted time courses of participants’ perception (Davis and Pope, 2002) and have been validated using an iPad-based continuous scale (Bird et al., 2016). This study shows that continuous ratings of two dimensions obtained using a 2D scale in an auditory task do not differ from those using 1D scales. This indicates that individuals are able to accurately rate two dimensions of complex stimuli simultaneously. This offers new possibilities of evaluating complex stimuli in real-time, especially if the associated perceptions are fluctuating in nature. Further studies with greater sample sizes are needed to fully validate the scale developed in this study.

Although the largest proportion of variance of the ratings in the visual and auditory tasks was explained by the changes in the physical values of the respective dimension, an effect of one dimension on the other was observed for the 2D as well as the 1D scales. For example, ratings of pitch were influenced by changes in sound pressure level. Using ‘snapshot’ ratings, it has previously been shown that multiple dimensions of an auditory stimuli, in particular frequency and sound pressure level, interact and impact response time, judgment and classification (Antinoro, 1969; Melara and Marks, 1990a,b; Neuhoff et al., 2002). These studies showed that stimuli were more accurately and rapidly evaluated when the two dimensions were congruent, e.g., high frequency—high sound pressure level. This might indicate that less cognitive effort is required when dimensions are congruent. Perhaps a consequence of this cognitive ease of congruency was observed in the present study: ratings in one dimension were influenced by the other dimension in a way that made them more similar. For example, an increase in sound pressure level led to higher ratings of pitch. These results are in line with previous research reporting an influence of one dimension on the evaluation of a second dimension in visual and auditory stimuli (Neuhoff et al., 1999; Suzuki and Takeshima, 2004; Walker et al., 2015). However, there was one exception: brighter circles were rated as smaller. Possibly, participants underestimated the size of the circles when the contrast between the circle and the background was low. In addition to the effects of the second dimension on the ratings of the first dimension, an effect of the physical attributes of the preceding stimulus on the ratings was observed for the visual task but not for the auditory task. This might demonstrate a learning effect because the auditory task was always performed after the visual task. If this was the case, it would be useful to investigate in future work whether a short training reduces this effect, in order to avoid a learning effect on the rating of stimuli of interest, e.g., pain and fatigue. Alternatively, it might indicate differences in the processing of visual and auditory stimuli.

Age has been shown to impact on the processing of sensory stimuli. Because of this, it was important to investigate whether older participants are similarly able to rate two dimensions of one stimulus simultaneously. Our results indicated no difference between younger and older participants that pertained to rating two dimensions simultaneously. In contrast, the effect of the size of the circle on the ratings of brightness as well as the effect of the physical dimension of sound frequency on the ratings of pitch were influenced by age. These findings are in line with known age effects on the perception of brightness (Spear, 1993; Sara and Faubert, 2000; Faubert, 2002) and high-frequency sounds (Weiss, 1963). Unlike previous literature suggesting that older participants underestimate the magnitude or the intensity of stimuli compared to younger participants (Heft and Robinson, 2014), younger participants in the present study rated brighter circles smaller than older participants. This discrepancy might be due to the difference of modality: Heft and Robinson used somatosensory and taste stimuli, while we used visual and auditory stimuli. Importantly, despite the observed age effects in the current study, younger and older participants were similarly able to rate the changes in the auditory and visual stimuli. The results of this study are limited to young (23–33 years of age) and older participants (62–76 years old). To confirm the ability of adult participants of all ages to rate on the 2D scale, studies involving participants between the ages of 33 and 62 years old need to be completed.

## 5. Conclusion

This study indicates that participants are able to simultaneously and continuously evaluate changes in two dimensions of visual and auditory stimuli using a 2D scale with rating accuracy not being different to 1D scales. Older participants were as able as younger participants to evaluate visual and auditory stimuli on the 2D scale, as well as auditory stimuli on the 1D scales.

## Data availability statement

The datasets presented in this study can be found in online repositories. The names of the repository/repositories and accession number(s) can be found below: https://github.com/ISR-lab.

## Ethics statement

The studies involving human participants were reviewed and approved by the Institutional Review Boards of The University of Utah and the Institutional Review Boards of the Salt Lake City Veteran’s Affairs Medical Center. The patients/participants provided their written informed consent to participate in this study.

## Author contributions

M-EH, RG, and PS contributed to the conception and design of the study. M-EH and TT contributed to the data collection and organization. M-EH, MR, and RG conducted the statistical analyses. M-EH and PS wrote the first draft of the manuscript. MA and M-EH provided the funding for this study. MA and AL provided the resources. All authors contributed to manuscript revision, read, and approved the submitted version.

## References

[B1] Allen E. J. Oxenham A. J. (2014). Symmetric interactions and interference between pitch and timbre. *J. Acoust. Soc. Am.* 135 1371–1379. 10.1121/1.4863269 24606275 PMC3985978

[B2] Antinoro F. (1969). Relation between sound intensity and amplitude of the AER at different stimulus frequencies. *J. Acoust. Soc. Am.* 46:1433. 10.1121/1.1911881 5361509

[B3] Bangalore S. Messerli F. H. (2006). Of statistical significance: “Trends” toward significance and optimism bias. *J. Am. Coll. Cardiol.* 48:1471. 10.1016/j.jacc.2006.07.011 17010814

[B4] Bird M.-L. Callisaya M. L. Cannell J. Gibbons T. Smith S. T. Ahuja K. D. (2016). Accuracy, validity, and reliability of an electronic visual analog scale for pain on a touch screen tablet in healthy older adults: A clinical trial. *Interact. J. Med. Res.* 5 e3–e12. 10.2196/ijmr.4910 26769149 PMC4731681

[B5] Davis K. D. Pope G. E. (2002). Noxious cold evokes multiple sensations with distinct time courses. *Pain* 98 179–185. 10.1016/s0304-3959(02)00043-x 12098630

[B6] De Gelder B. Bertelson P. (2003). Multisensory integration, perception and ecological validity. *Trends Cogn. Sci.* 7 460–467. 10.1016/j.tics.2003.08.014 14550494

[B7] Faubert J. (2002). Visual perception and aging. *Can. J. Exp. Psychol.* 56 164–176. 10.1037/h0087394 12271747

[B8] Garner W. R. (1976). Interaction of stimulus dimensions in concept and choice processes. *Cogn. Psychol.* 8:123. 10.1016/0010-0285(76)90006-2

[B9] Gracely R. H. Dubner R. (1987). Reliability and validity of verbal descriptor scales of painfulness. *Pain* 29 175–185. 3614956 10.1016/0304-3959(87)91034-7

[B10] Gracely R. H. McGrath P. Dubner R. (1978). Validity and sensitivity of ratio scales of sensory and affective verbal pain descriptors&colon; Manipulation of affect by diazepam. *Pain* 5 19–29. 10.1016/0304-3959(78)90021-0 673439

[B11] Heft M. W. Robinson M. E. (2014). Age differences in suprathreshold sensory function. *Age* 36 1–8. 10.1007/s11357-013-9536-9 23625154 PMC3889875

[B12] Kerrick J. S. Nagel D. C. Bennett R. L. (1969). Multiple ratings of sound stimuli. *J. Acoust. Soc. Am.* 45 1014–1017. 5791609 10.1121/1.1911487

[B13] Melara R. D. Marks L. E. (1990b). Interaction among auditory dimensions: Timbre, pitch, and loudness. *Percept. Psychophys.* 48 169–178. 10.3758/bf03207084 2385491

[B14] Melara R. D. Marks L. E. (1990a). Hard and soft interacting dimensions: Differential effects of dual context on classification. *Percept. Psychophys.* 47 307–325. 10.3758/bf03210870 2345683

[B15] Naumer M. J. Kaiser J. (2010). *Multisensory object perception in the primate brain.* Berlin: Springer Science & Business Media.

[B16] Neuhoff J. G. Kramer G. Wayand J. (2002). Pitch and loudness interact in auditory displays: Can the data get lost in the map?. *J. Exp Psychol. Appl.* 8 17–25. 10.1037//1076-898x.8.1.17 12009173

[B17] Neuhoff J. G. McBeath M. K. Wanzie W. C. (1999). Dynamic frequency change influences loudness perception: A central, analytic process. *J. Exp. Psychol. Hum. Percept. Perform.* 25 1050–1059. 10.1037/0096-1523.25.4.1050 10464944

[B18] Price D. D. McGrath P. A. Rafii A. Buckingham B. (1983). The validation of visual analogue scales as ratio scale measures for chronic and experimental pain. *Pain* 17 45–56. 6226917 10.1016/0304-3959(83)90126-4

[B19] Sara M. Faubert J. (2000). Aging, perception, and visual short-term memory for luminance-defined form. *Ophthalmic Physiol. Opt.* 20 314–322. 10962697

[B20] Smoorenburg G. F. (1970). Pitch perception of two-frequency stimuli. *J. Acoust. Soc. Am.* 48 924–942. 5480388 10.1121/1.1912232

[B21] Snyder J. S. Holder W. T. Weintraub D. M. (2009). Effects of prior stimulus and prior perception on neural correlates of auditory stream segregation. *Psychophysiology* 46 1208–1215. 10.1111/j.1469-8986.2009.00870.x 19674396

[B22] Spear P. D. (1993). Neural bases of visual deficits during aging. *Vis. Res.* 33 2589–2609. 8296455 10.1016/0042-6989(93)90218-l

[B23] Suzan E. Aviram J. Treister R. Eisenberg E. Pud D. (2015). Individually based measurement of temporal summation evoked by a noxious tonic heat paradigm. *J. Pain Res.* 8 409–415. 10.2147/jpr.s83352 26213476 PMC4509538

[B24] Suzuki Y. Takeshima H. (2004). Equal-loudness-level contours for pure tones. *J. Acoust. Soc. Am.* 116 918–933. 10.1121/1.1763601 15376658

[B25] Tabachnick B. G. Fidell L. S. (2013). *Using multivariate statistics.* Boston, MA: Pearson.

[B26] Vatakis A. Spence C. (2006). Audiovisual synchrony perception for music, speech, and object actions. *Brain Res.* 1111 134–142. 10.1016/j.brainres.2006.05.078 16876772

[B27] Walker P. Walker L. (2012). Size–brightness correspondence: Crosstalk and congruity among dimensions of connotative meaning. *Atten. Percept. Psychophys.* 74 1226–1240. 10.3758/s13414-012-0297-9 22484796

[B28] Walker P. Walker L. Francis B. (2015). The size-brightness correspondence: Evidence for crosstalk among aligned conceptual feature dimensions. *Attent. Percept. Psychophys.* 77 2694–2710. 10.3758/s13414-015-0977-3 26294420

[B29] Weiss A. D. (1963). Auditory perception in relation to age. *Hum. Aging Biol. Behav. Stud.* 111–140. 10.1037/10776-009

[B30] Woltman H. Feldstain A. MacKay J. C. (2012). An introduction to hierarchical linear modeling. *Tutor. Quant. Methods Psychol.* 8 52–69.

